# Comparative cation dependency of sugar transport by crustacean hepatopancreas and intestine

**DOI:** 10.1242/bio.20148904

**Published:** 2014-06-20

**Authors:** Ada Duka, Gregory A. Ahearn

**Affiliations:** Department of Biology, University of North Florida, Jacksonville, FL 32224, USA

**Keywords:** Atlantic lobster, *Homarus americanus*, Hepatopancreas, Intestine, BBMV, Brush border membrane vesicle, D-glucose, D-fructose, Na^+^-dependent, K^+^-dependent, Co-transport

## Abstract

Glucose is transported in crustacean hepatopancreas and intestine by Na^+^-dependent co-transport, while Na^+^-dependent D-fructose influx has only been described for the hepatopancreas. It is still unclear if the two sugars are independently transported by two distinct cation-dependent co-transporter carrier systems. In this study, lobster (*Homarus americanus*) hepatopancreas brush border membrane vesicles (BBMV) were used to characterize, in detail, the cation-dependency of both D-[^3^H]-glucose and D-[^3^H]-fructose influxes, while *in vitro* perfused intestines were employed to determine the nature of cation-dependent sugar transport across this organ. Over the sodium concentration range of 0–100 mM, both [^3^H]-glucose and [^3^H]-fructose influxes (0.1 mM; 1 min uptakes) by hepatopancreatic BBMV were hyperbolic functions of [Na^+^]. [^3^H]-glucose and [^3^H]-fructose influxes by hepatopancreatic BBMV over a potassium concentration range of 15–100 mM were hyperbolic functions of [K^+^]. Both sugars displayed significant (p<0.01) Na^+^/K^+^-dependent and cation-independent uptake processes. Transepithelial 25 µM [^3^H]-glucose and [^3^H]-fructose fluxes across lobster intestine over luminal sodium and potassium concentration ranges of 0–50 mM and 5–100 mM, respectively, were hyperbolic functions of luminal [Na^+^] and [K^+^]. As with hepatopancreatic sugar transport, transepithelial intestinal sugar transport exhibited both significant (p<0.01) Na^+^/K^+^-dependent and cation-independent processes. Results suggest that both D-glucose and D-fructose are transported by a single SGLT-type carrier in each organ with sodium being the “preferred”, high affinity, cation for both sugars in the hepatopancreas, and potassium being the “preferred”, high affinity, cation for both sugars in the intestine.

## INTRODUCTION

The SLC5 co-transporter gene family is a large family of 75 kDa proteins consisting of several sodium-dependent glucose co-transporter proteins (e.g. SGLT) that transport glucose and other solutes such as galactose across biological membranes in conjunction with the cations Na^+^ or H^+^ using transmembrane ion gradients to drive the co-transported substrate into the cytosol of both eukaryotic and prokaryotic cells (Turk and Wright, 1997). Four SGLTs have been experimentally described in detail: SGLT1, SGLT2, SGLT3, and SGLT4. SGLT1 is perhaps the best studied member and was the first cloned in 1987 (Hediger et al., 1987). SGLT1 functions as a high affinity, sodium-dependent glucose co-transporter and mainly participates in nutritional D-glucose and D-galactose absorption in the apical membrane of the mammalian intestine (Hediger et al., 1987). The SGLT1 transport protein consists of 14 transmembrane α-helices with both the N and C terminal facing the extracellular side of the membrane (Wright et al., 2007). SGLT2 is of low affinity, high capacity, and functions as a sodium-dependent glucose co-transporter as well, and is involved in renal and gastrointestinal transport of glucose (Kanai et al., 1994). SGLT 3 has been suggested to function as a glucose sensor rather than a glucose transporter (Díez-Sampedro et al., 2003). Within the last 10 years, a cDNA clone of SGLT4 was isolated from a human small intestine cDNA library and was reported to act as a mannose/1,5-anhydro-D-glucitol/fructose (Man/1,5AG/Fru) transporter in the intestine and kidney (Tazawa et al., 2005).

The SLC2 gene family consists of 50 kDa proteins that use a different mode of action than the SLC5 gene family. Sugars of this family are transferred across a membrane, in a sodium-independent manner, down the concentration gradient, by transport proteins that act as facilitated diffusion systems belonging to the GLUT family. Members of the GLUT family possess 12 transmembrane α-helices with both the N and C termini facing the intracellular side of the membrane (Brown, 2000). GLUT 2, located in the apical side of the intestinal epithelial cell, is the carrier protein responsible for the uptake of luminal glucose in the presence of high glucose concentrations. In addition, GLUT 2 is also present on the basolateral membrane and is responsible for transferring both glucose and fructose from the blood into the cytosol and for efflux of both sugars from cytoplasm to blood (Kellett and Helliwell, 2000; Caccia et al., 2007). GLUT 5 is a carrier protein specifically responsible for luminal fructose uptake (Caccia et al., 2007).

While sugar transporters have been studied in detail in mammals, very little is known about the absorption of sugars in invertebrates. The first study describing a detailed model for carrier-mediated sugar transport across an insect epithelium was published in 2007 (Caccia et al., 2007). These authors used apical and basolateral fluxes of radiolabelled D-glucose and D-fructose, immunocytochemistry, and Western blot analysis to describe the sugar carrier proteins in larval parasitoid wasp (*Aphidius ervi*, Hymenoptera) midgut epithelial cells. The localization of SGLT1-like and GLUT5-like transporters were shown to be present on the intestinal brush-border membrane, while GLUT2-like proteins were present on the basolateral side of the gut and the brush-border membrane (Caccia et al., 2007), similar to the arrangement of these sugar carrier proteins in mammalian intestine (Uldry and Thorens, 2004).

The crustacean hepatopancreas has several functions including synthesis and secretion of digestive enzymes, absorption of nutrients, secretion of emulsifiers, and storage of carbohydrates and metals. The hepatopancreas epithelium is lined with at least four different cell types (Verri et al., 2001). The four cells types each have different structures, which provide different roles in digestive and absorptive functions (Wright and Ahearn, 1997; Verri et al., 2001). The crustacean intestine runs from the posterior portion of the pyloric foregut to the anterior part of the chitinized hindgut and is comprised of a single epithelial cell type possessing a microvillar apical border. The intestine is considered to be a scavenger organ, absorbing leftover nutrients after hepatopancreatic function (Wright and Ahearn, 1997).

Previous studies have shown glucose transport in both crustacean organs by facilitative transport and Na^+^-dependent co-transport (Ahearn et al., 1985; Obi et al., 2011), while Na^+^-dependent D-fructose influx has only been described for the hepatopancreas (Sterling et al., 2009). In neither organ have the details of ion-dependent sugar transport been elucidated and it is still unclear as to whether the two sugars are independently transported by two distinct co-transporter carrier systems or by the same transporter. In the present study, lobster (*Homarus americanus*) hepatopancreatic brush-border membrane vesicles (BBMV) were used to characterize, in detail, the cation-dependency of both [^3^H]-glucose and [^3^H]-fructose influxes, while *in vitro* perfused intestines were employed to determine cation-dependent sugar transport in this organ. Results suggested that both D-glucose and D-fructose are transported by a single carrier process that resembles SGLT4 in each organ with sodium being the “preferred”, high affinity, cation for both sugars in the hepatopancreas, and potassium being the “preferred”, high affinity, cation for both sugars in the intestine.

## RESULTS

### Hepatopancreas

#### Effect of freezing on the sugar transport activity of lobster hepatopancreatic BBMV

**Fig. 1.**
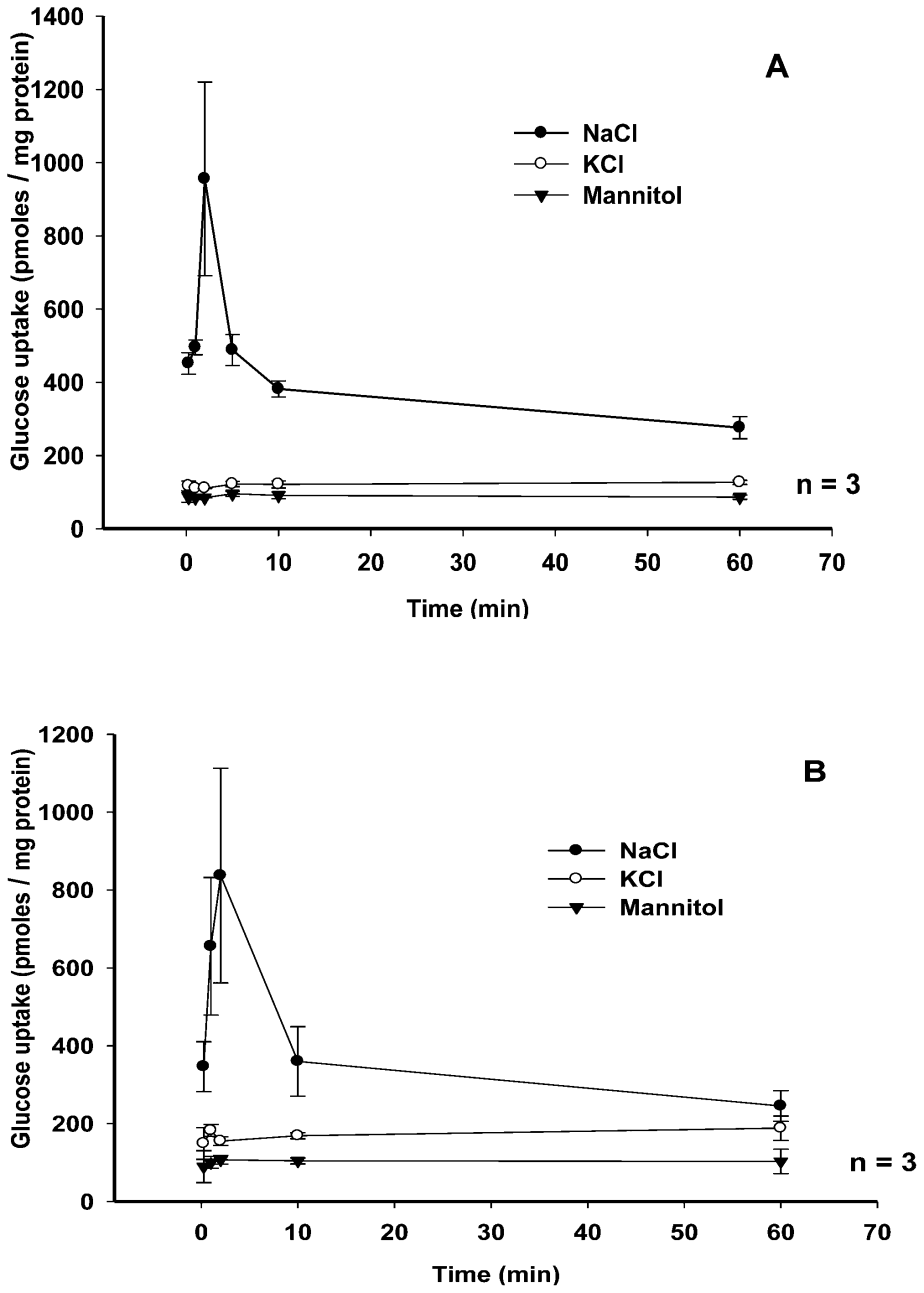
Frozen BBMV act in a similar manner to fresh BBMV. (A) Time course of 0.1 mM [^3^H]-glucose uptake by fresh lobster hepatopancreatic brush-border membrane vesicles (BBMV) loaded with 300 mM mannitol, 12 mM Hepes/Tris at pH 7.0 and incubated in either 150 mM NaCl, 150 mM KCl or 300 mM mannitol each with 12 mM Hepes/Tris pH 7.0 for periods of time from 15 s to 60 min. (B) Time course of 0.1 mM [^3^H]-glucose uptake by frozen BBMV with the same experimental conditions as panel A. The experiment was conducted three times (3 lobsters) with 3 replicates/treatment. Symbols are means ± 1 SEM.

To assess the effect of freezing on the transport activity of lobster hepatopancreatic brush border membrane vesicles (BBMV), vesicles prepared from an individual lobster hepatopancreas were loaded with 300 mM mannitol, 12 mM Hepes/Tris at pH 7.0. Results in Fig. 1A,B indicate the sugar uptake by both fresh and frozen vesicles in NaCl medium displayed an initial uptake overshoot followed by a slow return to similar equilibrium values at 60 min. The overshoot displayed in both the fresh and frozen vesicles was triple its respective equilibrium value. Sugar uptake by both fresh and frozen vesicles displayed no significant uptake overshoot in media containing potassium or mannitol. The similarity of these data suggest that lobster hepatopancreatic BBMV can be frozen and when used will yield similar uptake values as vesicles used immediately after protein isolation. All vesicle data beyond this point have been obtained from frozen BBMV.

#### Increasing NaCl concentrations stimulate glucose and fructose influx by BBMV in a hyperbolic manner

**Fig. 2.**
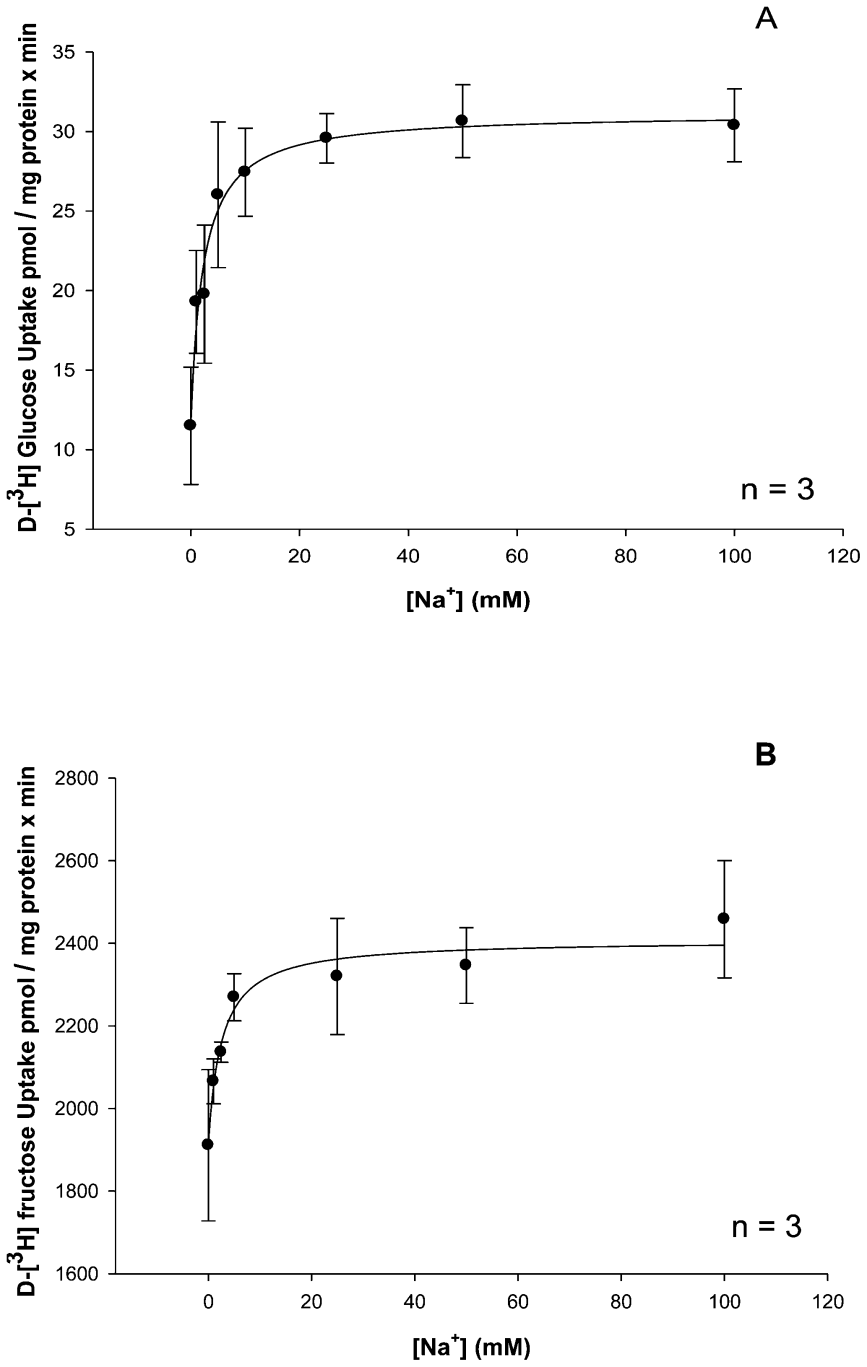
Increasing NaCl concentrations stimulate glucose and fructose transport in a hyperbolic manner. Effect of increasing Na^+^ concentrations on 1 min 0.1 mM [^3^H]-glucose (A) and 0.1 mM [^3^H]-fructose (B) uptake in lobster hepatopancreatic BBMV. Vesicles were loaded with 200 mM mannitol, 12 mM Hepes/Tris at pH 7.0 and incubated in various NaCl concentrations (0, 1, 2.5, 5, 10, 25, 50, 100 mM), 12 mM Hepes/Tris at pH 7.0. The experiment was conducted three times with 5 replicates/treatment. Symbols are means ± 1 SEM. Curve fit lines and resulting kinetic constant values were obtained using Sigma plot 10.0 software. Kinetic constants are displayed in Table 1.

To assess the effect of increasing NaCl concentrations on D-glucose and D-fructose influx kinetics by hepatopancreatic BBMV, vesicles were loaded with 200 mM mannitol, 12 mM Hepes/Tris at pH 7.0 and were incubated for 1 min in external media containing 0.1 mM [^3^H]-glucose or 0.1 mM [^3^H]-fructose, increasing concentrations of sodium (0, 1, 2.5, 5, 10, 25, 50, 100 mM NaCl) and 12 mM Hepes/Tris at pH 7.0. Results in Fig. 2 display influx as a hyperbolic function of [Na^+^], which followed the Michaelis–Menten equation for carrier-mediated transport:
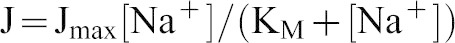
(1)where J is [^3^H]-glucose or [^3^H]-fructose influx (ρmol/mg protein × min), J_max_ is maximal glucose or fructose influx rate (ρmol/mg protein × min), K_M_ is the apparent affinity binding constant (mM), and [Na^+^] is NaCl concentration in mM. Fig. 2A,B indicate that NaCl was able to stimulate the uptake of both sugars in a saturable manner, indicating significant (p<0.01) Na^+^-dependent and Na^+^-independent uptake processes (vertical axis intercepts). The resulting K_M_ values for glucose and fructose as shown in Table 1 were similar (2.30±0.59 and 2.58±0.95 mM, respectively). The maximal transport rates, however, were different, with transport being 26 times faster for fructose than for glucose.

**Table 1.**
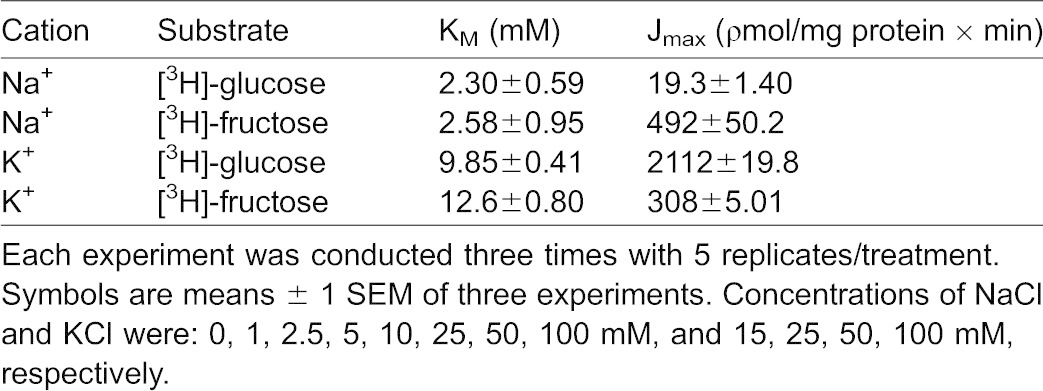
Effect of increasing NaCl and KCl concentrations on 0.1 mM [^3^H]-glucose or 0.1 mM [^3^H]-fructose influx kinetic constants in lobster hepatopancreas

#### Increasing KCl concentrations stimulate glucose and fructose influx by BBMV in a hyperbolic manner

**Fig. 3.**
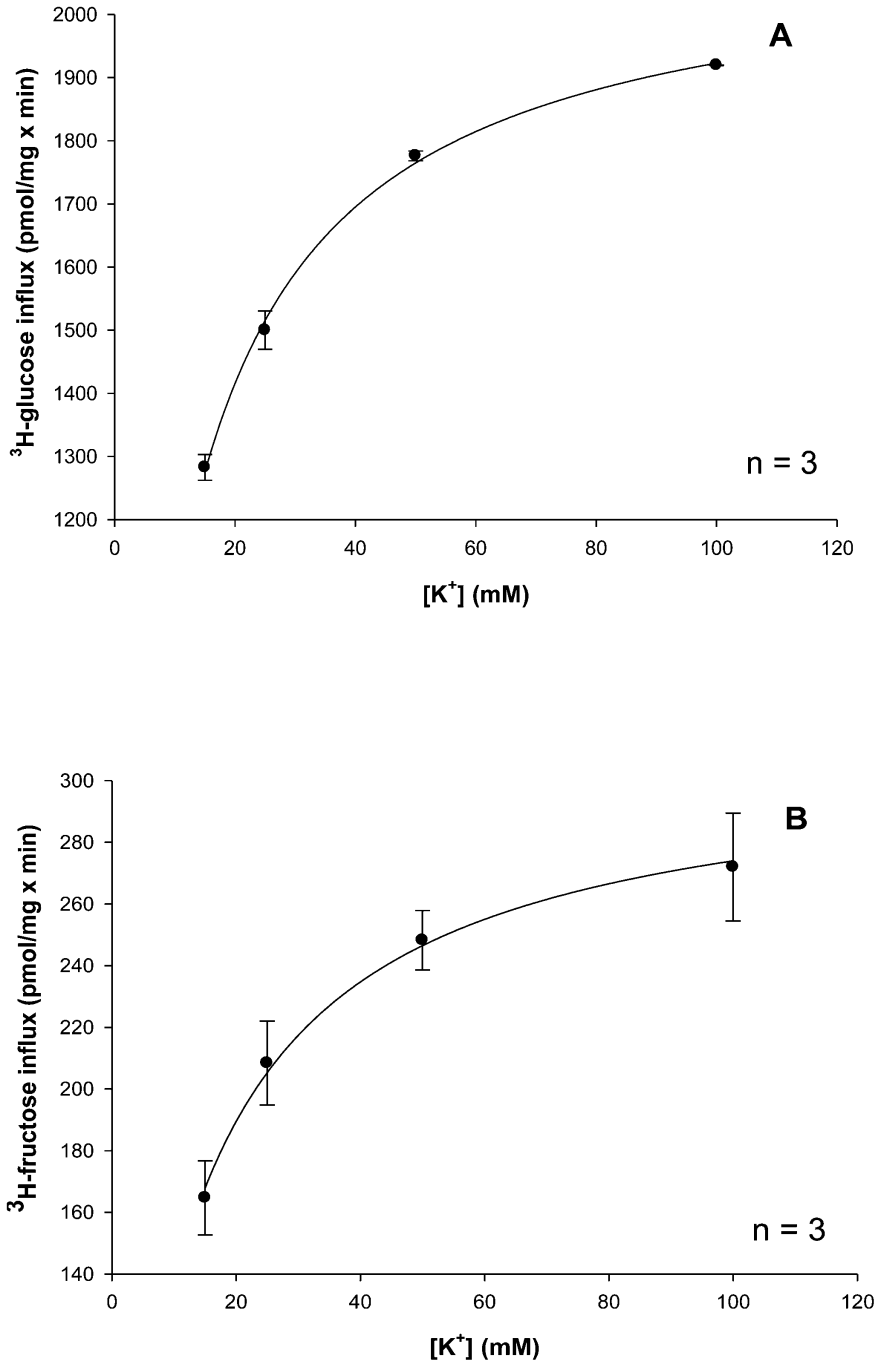
Increasing KCl concentrations stimulate glucose and fructose influx in a hyperbolic manner. Effect of increasing K^+^ concentrations on 1 min 0.1 mM [^3^H]-glucose (A) and 0.1 mM [^3^H]-fructose (B) uptake in lobster hepatopancreatic BBMV. Vesicles were loaded with 200 mM mannitol, 12 mM Hepes/Tris at pH 7.0 and incubated in various KCl concentrations (15, 25, 50, 100 mM), 12 mM Hepes/Tris at pH 7.0. The experiment was conducted three times with 5 replicates/treatment. Symbols are means ± 1 SEM. Curve fit lines and resulting kinetic constant values were obtained using Sigma plot 10.0 software. Kinetic constants are displayed in Table 1.

There is not much research on the effect of potassium on sugar influx since adult lobsters are mostly carnivores, consuming little potassium in the foods they eat. This experiment was designed to demonstrate whether or not potassium was an effective symporter for glucose and fructose transport. Fig. 3A,B illustrates the effect of various potassium concentrations (5, 15, 25, 50 and 100 mM KCl) on 1 min uptake values for 0.1 mM [^3^H]-glucose and 0.1 mM [^3^H]-fructose by hepatopancreatic BBMV. Both D-glucose and D-fructose influxes were hyperbolic functions of [K^+^] and followed the Michaelis–Menten equation (Eqn 1). Significant (p<0.01) K^+^-independent D-glucose and D-fructose uptake processes were also observed by extrapolation (vertical axis intercepts). The resulting K_M_ values for glucose and fructose influxes in potassium medium as shown in Table 1 appear to be similar (9.85±0.4 and 12.6±0.80 mM, respectively). The maximal transport rates, however, were different, with transport being 7 times faster for glucose than for fructose. Table 1 also suggests that the apparent cation-binding affinity was greater (lower K_M_) in external NaCl than in KCl, indicating that Na^+^ might be the “preferable ion” for stimulating hepatopancreatic BBMV glucose and fructose uptake in the American lobster.

#### Effects of D-fructose on the kinetics of [^3^H]-glucose influxes by BBMV in NaCl incubation medium

**Fig. 4.**
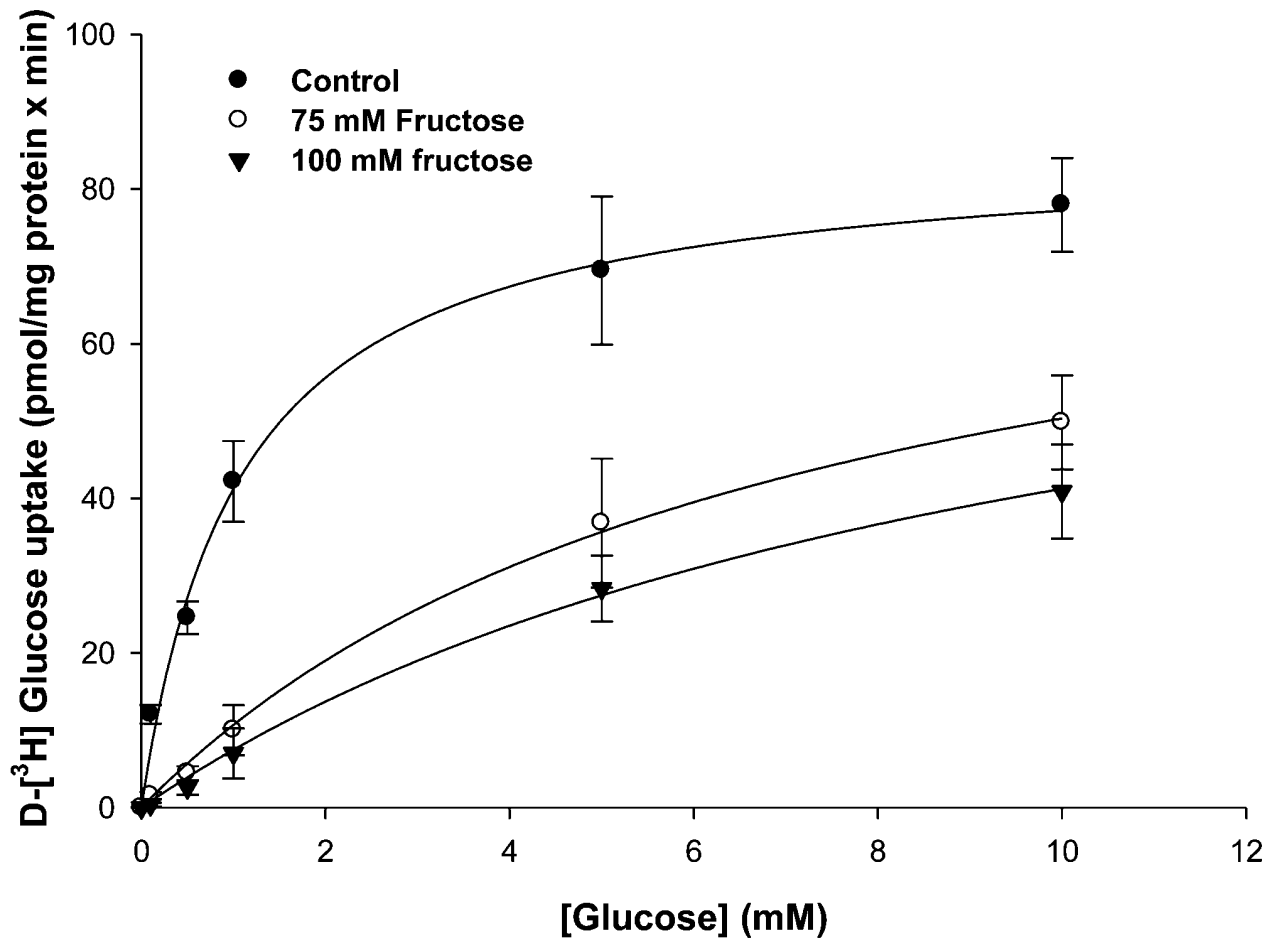
Effect of increasing D-[^3^H]-glucose concentrations (0.1, 0.5, 1, 5, 10 mM) on 1 min uptake in NaCl incubation medium in the presence and absence of D-fructose (75, 100 mM). Vesicles were loaded with 310 mM mannitol, 12 mM Hepes/Tris at pH 7.0 and incubated in 100 mM NaCl, 12 mM Hepes/Tris at pH 7.0. Appropriate quantities of mannitol were added to external media to keep them osmotically equivalent to internal medium. The experiment was conducted three times with 5 replicates/treatment. Symbols are means ± 1 SEM. Curve fit lines and resulting kinetic constant values were obtained using Sigma Plot 10.0 software. Kinetic constants are displayed in Table 2.

[^3^H]-glucose influx (1 min uptakes) as a function of external [D-glucose] was measured in NaCl incubation medium in the presence and absence of D-fructose as described by Segel (Segel, 1975) (Fig. 4). Glucose influx under control conditions (lacking D-fructose) was a hyperbolic function of [D-glucose] and followed the Michaelis–Menten equation (Eqn 1) for carrier-mediated transport, where J is unidirectional [^3^H]-glucose influx (ρmol/mg protein × min), J_max_ is the maximal influx rate, K_M_ is an apparent affinity binding constant (mM), and [Glu] is the external sugar concentration (mM).

[^3^H]-glucose influx kinetics observed in the presence of 75 mM and 100 mM D-fructose in the external incubation medium are also displayed in Fig. 4 for comparison with influx under control conditions. In the presence of D-fructose, influxes of [^3^H]-glucose at each external [D-glucose] were lower than those occurring at the same [D-glucose] in the absence of D-fructose. A slightly greater reduction was seen in 100 mM D-fructose than 75 mM D-fructose. Table 2 indicates that the addition of D-fructose to the external medium led to an increase in [^3^H]-glucose influx K_M_, but had no effect on [^3^H]-glucose influx J_max_. These results suggest that [^3^H]-glucose influx in NaCl incubation medium occurred by a carrier-mediated transport process that appeared to be competitively inhibited by D-fructose.

**Table 2.**
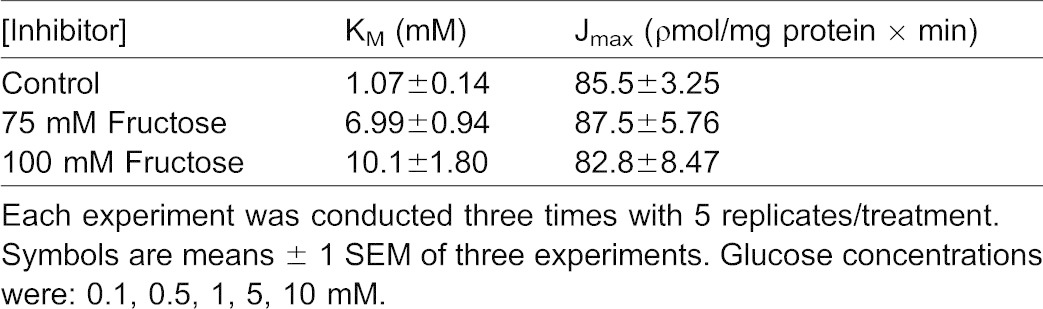
Kinetic constants showing the effect of increasing glucose concentrations on [^3^H]-glucose transport by BBMV in medium containing 100 mM NaCl in the presence and absence of various inhibitor concentrations

### Intestine

#### NaCl stimulates glucose and fructose intestinal transepithelial transport in a hyperbolic manner

**Fig. 5.**
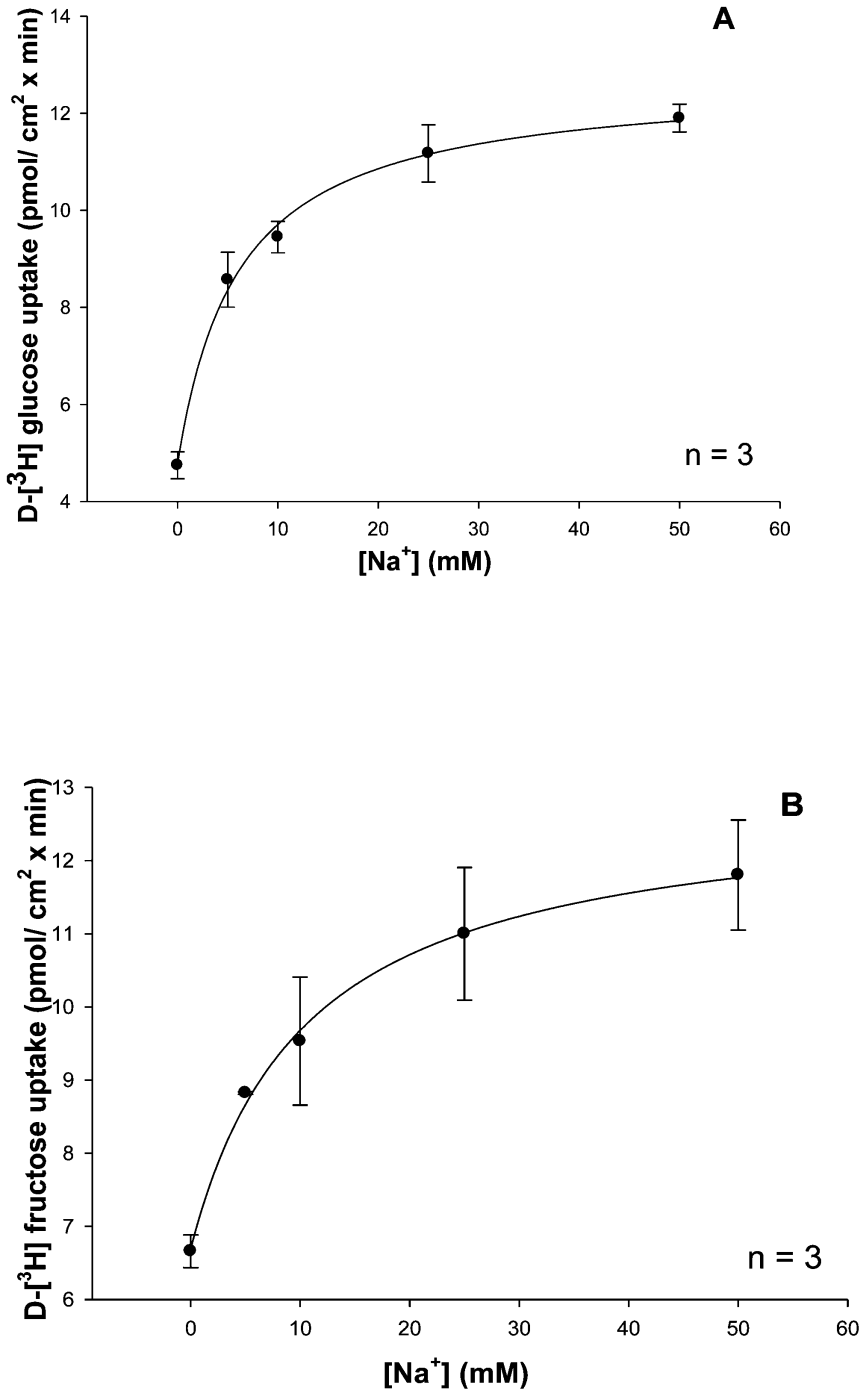
Effect of increasing sodium concentrations (0, 5, 10, 25, 50 mM) on intestinal mucosal to serosal (MS) transmural 25 µM D-[^3^H]-glucose (A) and D-[^3^H]-fructose (B) transport. Each experiment was conducted three times with 3 replicates/treatment. Symbols are means ± 1 SEM. Curve fit lines and resulting kinetic constant values were obtained using Sigma plot 10.0 software. Kinetic constants are displayed in Table 3.

To assess the effect of increasing sodium concentrations on D-glucose and D-fructose transepithelial transport, mucosal to serosal (MS) transport experiments over a 30 min time course at a variety of sodium concentrations (0, 5, 10, 25, 50 mM) with 25 µM [^3^H]-glucose or 25 µM [^3^H]-fructose were performed in triplicate (three animals) using perfused intestines. Slopes of the time course data were determined by linear regression analysis and data curve fitting procedures using Sigma Plot 10.0 software. Results in Fig. 5A,B shows that increasing concentrations of luminal NaCl increased [^3^H]-glucose and [^3^H]-fructose transport in a hyperbolic manner, indicating significant (p<0.05) sodium-dependent and sodium-independent (vertical axis intercepts) uptake processes. Each data point represents the uptake slope ± SEM at each concentration of sodium. The resulting K_M_ values for glucose and fructose in sodium medium as shown in Table 3 appear to be quite similar suggesting that both sugars may be transported by a single carrier process in the intestine with a 3-fold lower binding affinity than in the hepatopancreas (Tables 1 and 3).

**Table 3.**
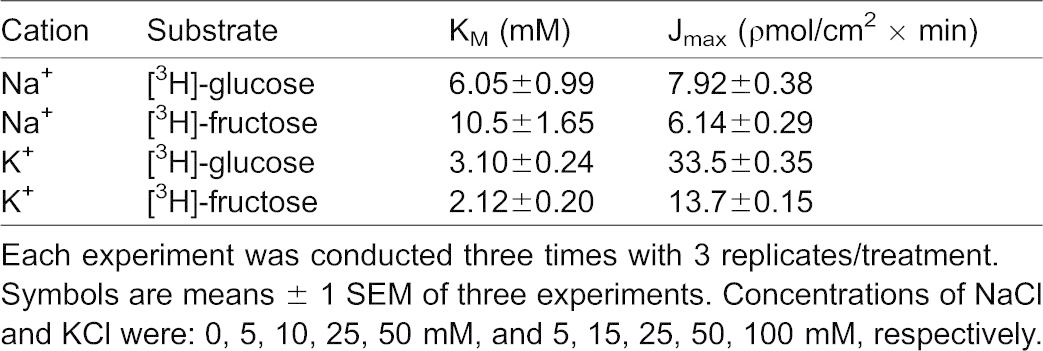
Effect of increasing NaCl and KCl concentrations on 25 µM [^3^H]-glucose or 25 µM [^3^H]-fructose transepithelial transport kinetic constants in lobster intestine

#### KCl stimulates glucose and fructose intestinal transepithelial transport in a hyperbolic manner

**Fig. 6.**
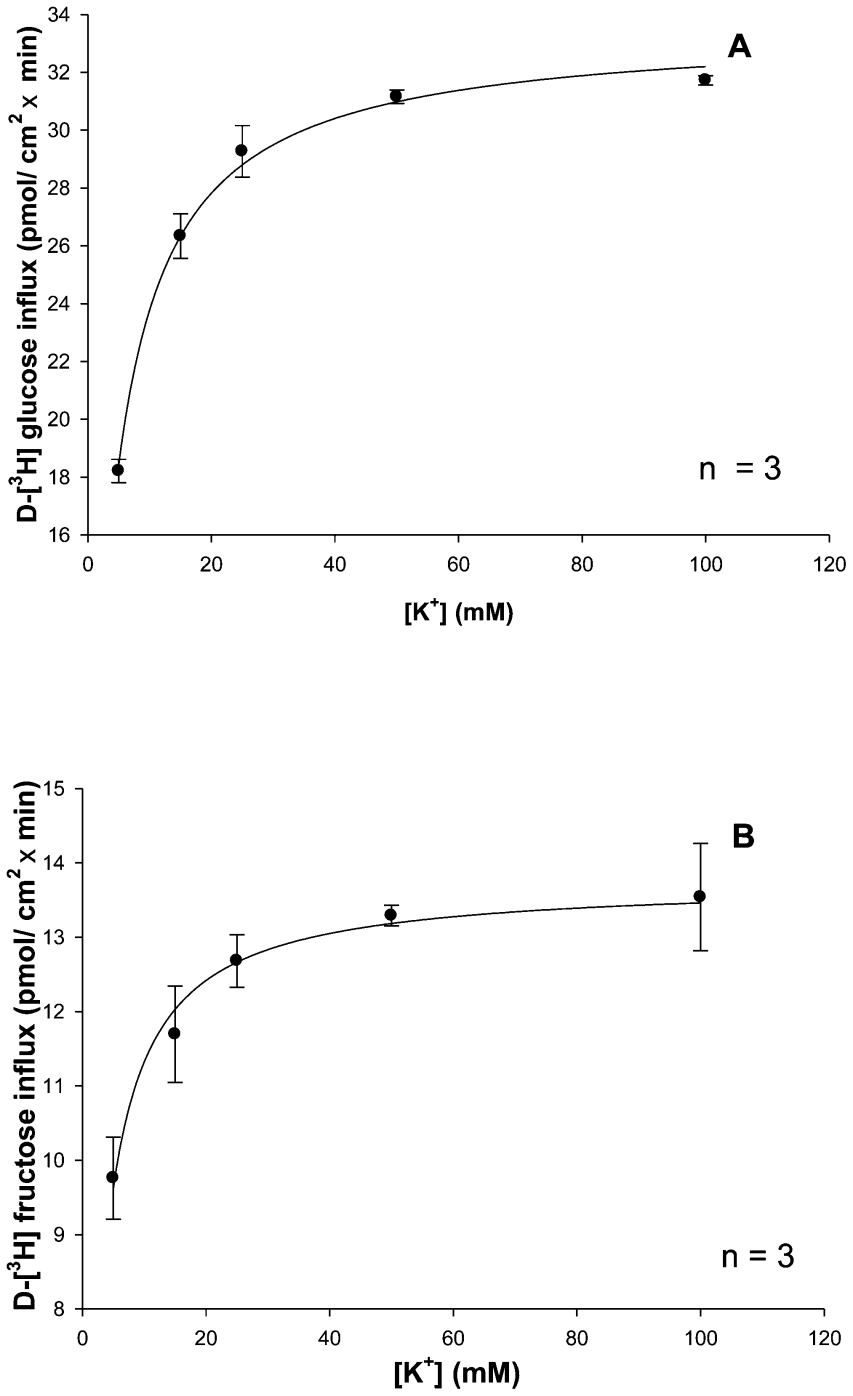
Effect of increasing potassium concentrations (5, 15, 25, 50, 100 mM) on intestinal mucosal to serosal (MS) transmural 25 µM D-[^3^H]-glucose (A) and D-[^3^H]-fructose (B) transport. The experiment was conducted three times with 3 replicates/treatment. Symbols are means ± 1 SEM. Curve fit lines and resulting kinetic constant values were obtained using Sigma plot 10.0 software. Kinetic constants are displayed in Table 3.

In the intestine, transepithelial transport experiments over a 30 min time course at a variety of potassium concentrations (5, 15, 25, 50 and 100 mM KCl) with 25 µM [^3^H]-glucose and [^3^H]-fructose were performed in triplicate (three animals each) (Fig. 6A,B). As in the hepatopancreas, results in Fig. 6A,B shows that increasing concentrations of luminal KCl increased transepithelial [^3^H]-glucose and [^3^H]-fructose transport in a hyperbolic manner indicating significant (p<0.05) potassium-dependent uptake processes. Each data point represents the uptake slope ± SEM at each concentration of potassium. The resulting K_M_ values for glucose and fructose in potassium medium as shown in Table 3 appear to be quite similar suggesting a single carrier process in the intestine. Table 3 also suggests that the apparent cation-binding affinity was greater (lower K_M_) in external KCl than in NaCl, indicating that K^+^ might be the “preferable ion” for stimulating glucose and fructose uptake in the intestine of the American lobster.

#### Effects of D-fructose on the kinetics of transepithelial [^3^H]-glucose transport in KCl incubation medium

**Fig. 7.**
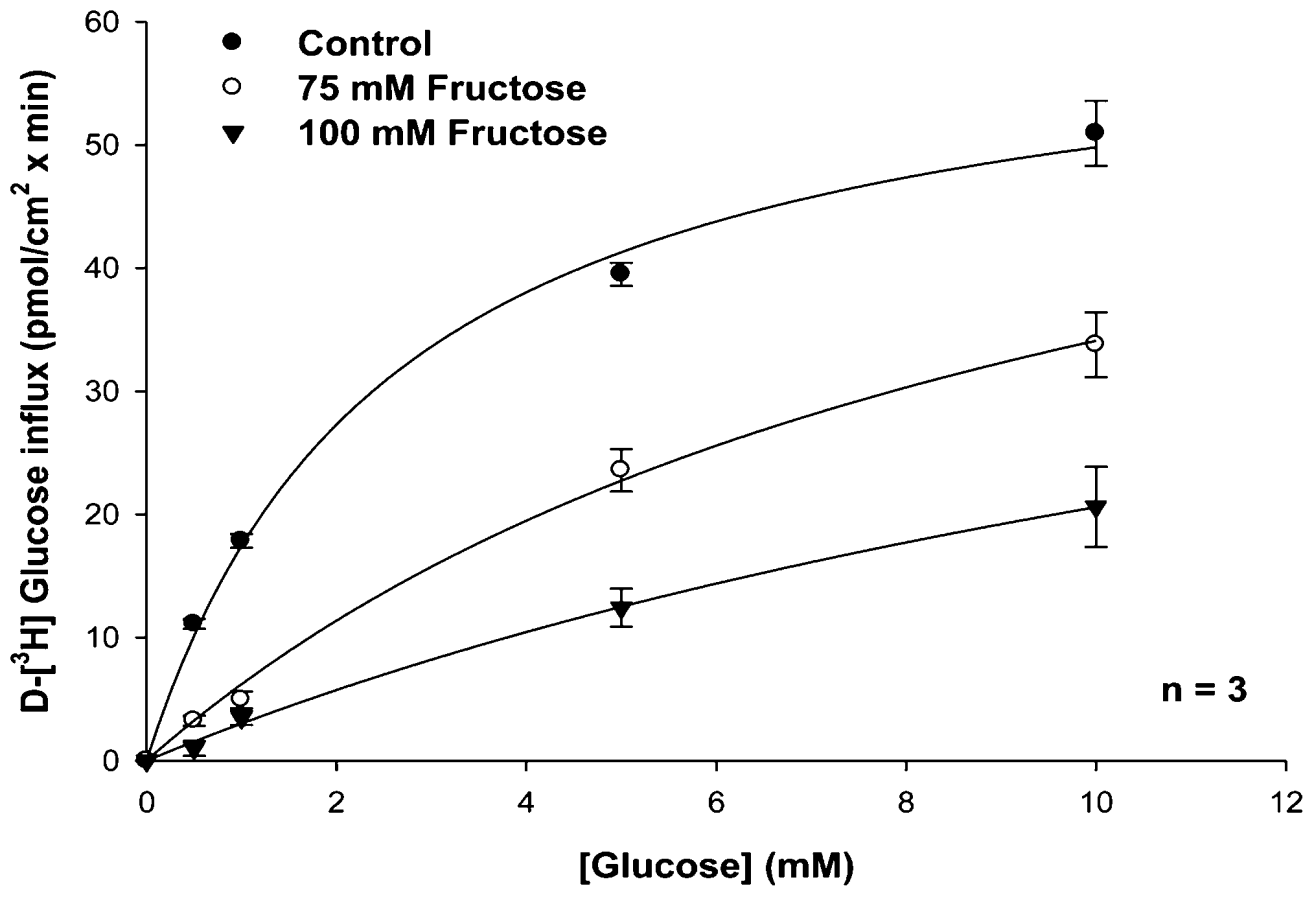
Effect of increasing mucosal D-[^3^H]-glucose concentrations (0.5, 1, 5, 10 mM) on transmural MS glucose transport in KCl incubation medium in the presence and absence of D-fructose (75, 100 mM). The experiment was conducted three times with 3 replicates/treatment. Symbols are means ± 1 SEM. Curve fit lines and resulting kinetic constant values were obtained using Sigma Plot 10.0 software. Kinetic constants are displayed in Table 4.

Transepithelial [^3^H]-glucose transport as a function of external [D-glucose] was measured in KCl incubation medium in the presence and absence of D-fructose (Segel, 1975) (Fig. 7). Glucose influx under control conditions (lacking D-fructose) was a hyperbolic function of [D-glucose] and followed the Michaelis–Menten equation (Eqn 1) for carrier-mediated transport. [^3^H]-glucose influx kinetics observed in the presence of 75 mM and 100 mM D-fructose in the mucosal medium are also displayed in Fig. 7 for comparison with influx under control conditions. In the presence of D-fructose, influxes of [^3^H]-glucose at each D-glucose concentration were lower than those occurring at the same [D-glucose] in the absence of D-fructose. A greater reduction was seen in 100 mM D-fructose than 75 mM D-fructose. Table 4 indicates that the addition of D-fructose to the mucosal medium led to an increase in [^3^H]-glucose influx K_M_, but had no effect on [^3^H]-glucose influx J_max_. These results suggest that [^3^H]-glucose influx in KCl incubation medium occurred by a carrier-mediated transport process that appeared to be competitively inhibited by D-fructose in the intestine.

**Table 4.**
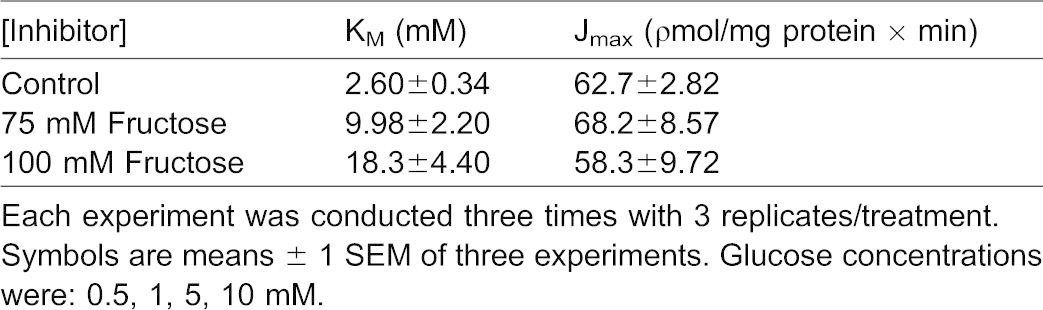
Kinetic constants showing the effect of increasing glucose concentrations on transepithelial [^3^H]-glucose transport in medium containing 100 mM KCl in the presence and absence of various inhibitor concentrations

## DISCUSSION

The results of the present investigation suggest that both D-glucose and D-fructose are transported across the intestinal epithelium and hepatopancreatic brush border membrane of the American lobster (*Homarus americanus*), at least in part, by a shared Na^+^/K^+^-stimulated transport process with properties resembling those of a mammalian SGLT-type sugar carrier.

The present research has examined the effects of a freezing preservation technique on lobster hepatopancreatic BBMV glucose transport characteristics. Previous experiments on the preservation of glucose transport and enzyme activities during freezing of BBMV from the intestinal epithelium of the warm-water euryhaline fish, *Oreochromis mossambicus*, showed that the freezing of BBMV in liquid nitrogen preserved the properties of glucose transport found in fresh vesicles, including sodium-dependency, and overshoot characteristics (Reshkin et al., 1988). Long-term storage and maintenance of functionality of a membrane system offers advantages over exclusive use of fresh protein preparations. The similarity in activity between frozen and fresh preparations greatly reduces experimental time and thus greatly increases productivity. Additionally, preservation of a single large vesicle preparation permits an increased number of experiments to be conducted on the same vesicle population, thereby reducing the variability created due to organismal and preparative differences. In this investigation, however, experiments were conducted with vesicles prepared from individual lobster hepatopancreases and were repeated three times (3 lobsters). Rapid freezing in liquid nitrogen and storage in −80°C (at least 48 hours), in combination with slow, gentle thawing of the samples on ice, resulted in a sample preparation with essentially identical glucose transport characteristics to those found in fresh preparations. The overshoot displayed in both the fresh and frozen vesicle preparations was triple its respective equilibrium value. Glucose uptake by both fresh and frozen vesicle preparations displayed no significant uptake overshoot in media containing potassium or mannitol. The overshoot phenomenon was thus conserved and essentially unchanged in glucose transport by frozen vesicle preparations. The slow thawing appears to result in less structural and functional damage to membrane proteins, resulting in an improved preservation of original transport properties (Rudolph and Crowe, 1985; Reshkin et al., 1988). Thus, slow thawing on ice may be an essential component for freeze–thaw vesicle preservation. All hepatopancreatic BBMV data in this investigation were obtained using at least 3 sets of pooled frozen BBMV preparations.

Glucose transport has been shown to be sodium-dependent in the invertebrate digestive tract (Ahearn and Maginniss, 1977; Ahearn et al., 1985; Blaya et al., 1998; Verri et al., 2001; Vilella et al., 2003). Glucose uptake in the absence of sodium was equilibrative (Ahearn et al., 1985). Sodium-coupled D-glucose transport by digestive tract epithelial cells was confirmed in the present study. While Na^+^-dependent D-glucose influx has been extensively investigated, Na^+^-dependent D-fructose influx has only been described in the hepatopancreas (Sterling et al., 2009). The observed sodium-dependent fructose influx by BBMV is in contrast to the mammalian paradigm, in which fructose uptake by BBMV involves a Na^+^-independent GLUT-like facilitative transporter. Mucosal GLUT 5 is a known fructose transporter previously identified in the mammalian intestinal epithelial brush-border membrane (Miyamoto et al., 1994; Corpe et al., 2002). Increasing concentrations of sodium stimulated D-fructose influx by lobster BBMV in a hyperbolic manner, similar to sodium-coupled D-glucose transport by BBMV (Fig. 2). Similarity in K_M_ values for Na^+^-dependent D-glucose and D-fructose transport by hepatopancreatic BBMV (Table 1) suggest that they may be transported by a single carrier process with substrate specificities different from those demonstrated by mammalian SGLT1. The maximal transport rate, however, was different, with transport of fructose being 25 times faster than for glucose (Table 1). The sodium-dependent fructose transport activity reported here can be compared with that of mammalian SGLT4, which is known to transport fructose, in addition to glucose, in a sodium-dependent manner (Tazawa et al., 2005). D-fructose (75 and 100 mM) inhibited D-glucose influx in a competitive manner (Table 2). Increasing [D-fructose] led to an increase in K_M_ (decrease in binding affinity) but no change in J_max_. D-glucose and D-fructose appeared to be competing for the same binding site on this sodium-coupled hexose transporter in the hepatopancreas.

Both D-glucose and D-fructose influxes were hyperbolic functions of the potassium concentration (Fig. 3) and followed the Michaelis–Menten equation (Eqn 1). The resulting K_M_ values for glucose and fructose transport in potassium medium appeared to be similar (Table 1). The maximal transport rates, however, were different with transport being 7 times faster for glucose than for fructose. Both D-glucose and D-fructose appear to be transported by a single carrier process with sodium and potassium as alternative driving cations. The apparent cation-binding affinity was approximately 5 times greater (lower K_M_) in external NaCl than in KCl, indicating that Na^+^ might be the “preferable ion” for stimulating hepatopancreatic BBMV glucose and fructose uptake in the American lobster. While D-glucose transport, using transmembrane gradients of either sodium or potassium as driving forces, have been previously described for the Atlantic white shrimp (Simmons et al., 2012; Obi et al., 2013), sodium or potassium-coupled D-fructose transport by lobster hepatopancreatic BBMV has only been reported in the present investigation. The question, however, arises as to why increasing KCl concentrations stimulate sugar influx by BBMV in a hyperbolic manner as shown in the present investigation (Fig. 3), while in a previous study by Ahearn et al. a transmembrane potassium gradient was unable to result in a [^3^H]-glucose uptake overshoot in the same species (Ahearn et al., 1985). A likely answer is that potassium may be acting as an activator rather than a driver ion mediating D-glucose transport across the hepatopancreatic brush border membrane. The binding of K^+^ to cation-dependent sugar transporters may open available binding sites for sugars that would have been closed prior to cation binding. Sugar transport would therefore be driven by the concentration gradient of the sugar itself without the use of a driving ion.

*In vitro* perfused intestines were employed to characterize the nature of cation-dependent D-glucose and D-fructose transport in this organ. Sodium-dependent D-glucose co-transport has been reported in both hepatopancreas and intestine (Ahearn et al., 1985; Obi et al., 2011), while sodium-dependent D-fructose co-transport has only previously been described for the hepatopancreas (Sterling et al., 2009). In the present study, however, sodium-dependent transepithelial D-fructose transport was disclosed. Increasing concentrations of sodium stimulated D-fructose intestinal epithelial transport in a hyperbolic manner, similar to sodium-coupled D-glucose transport in the same organ (Fig. 5). Similarity in K_M_ values for Na^+^-dependent D-glucose and D-fructose transport suggest that they were transported by a single carrier process in the intestine (Table 3). Furthermore, D-fructose (75 and 100 mM) inhibited D-glucose transport in a competitive manner (Fig. 7), suggesting that D-glucose and D-fructose appeared to be competing for the same binding site on a shared hexose transporter.

Potassium-dependency of glucose and fructose transport was investigated in lobster intestine in the present study. No previous studies have reported K^+^-dependent sugar transport in any crustacean intestine. In a recent study, D-glucose and D-fructose transport were examined in the lobster intestine, but only sodium was used as the cation driving force (Obi et al., 2011). In lobster, both D-glucose and D-fructose intestinal epithelial transport were hyperbolic functions of potassium concentration (Fig. 6) and followed the Michaelis–Menten equation (Eqn 1). The resulting K_M_ values for D-glucose and D-fructose transport in potassium medium appeared to be similar (Table 3). The maximal transport rates, however, were different with transport being 2.5 times faster for glucose than for fructose. Both D-glucose and D-fructose appeared to be transported by a single carrier process with sodium and potassium as alternative co-transported cations. The apparent cation-binding affinity was approximately 5 times greater (lower K_M_) in external KCl than in NaCl (Table 3), indicating that K^+^ might be the “preferable ion” for stimulating intestinal epithelial glucose and fructose transport in the American lobster.

The magnitudes of the potassium concentration gradient or membrane potential across the epithelial luminal membrane *in vivo* is not known, but even though the American lobster is mostly carnivorous, they do include some amount of marine algae in their diets (Conklin, 1995), which are high in potassium. An increase in luminal potassium concentration would disturb the potassium equilibrium across the epithelial luminal membrane and result in an increased inwardly-directed electrochemical driving force for the uptake of potassium from lumen to epithelial cytosol (Obi et al., 2013). An estimation of the minimum luminal [K^+^] needed to stimulate sugar uptake by an *in vivo* electrochemical driving force has been previously calculated using the potassium Nernst equation and was reported to be values greater than 10 mM (as occurs in seawater) (Obi et al., 2013).

In addition to cation-dependent glucose and fructose carrier processes observed in the hepatopancreas and intestine, sodium-independent glucose and fructose transport processes appeared to be observed as well. In the absence of sodium or potassium (0 mM; vertical axis intercepts), there was still a considerable amount of glucose and fructose uptake by hepatopancreatic BBMV and intestinal transepithelial transport (Figs 2, 3, 5, 6), suggesting the possible presence of lobster GLUT2-like and GLUT5-like transporters, respectively. In a recent study by Sterling et al. polyclonal antibodies to mammalian GLUT2 and GLUT5 were used to determine the localization of orthologous lobster hepatopancreas proteins by western blot analysis (Sterling et al., 2009). Both GLUT2 and GLUT5 were found in the hepatopancreas and intestine (Sterling et al., 2009). In addition, GLUT5-like transporters were localized to the brush border membrane of lobster intestine using immunohistochemical methods (Obi et al., 2011). Furthermore, western blot analysis using a rabbit anti-human SGLT4 antibody located an SGLT4-like protein in the lobster hepatopancreas (Sterling et al., 2009). These findings, and those reported in the present investigation, suggest that the mammalian epithelial sugar transporter ensemble (e.g. SGLT1, GLUT2, GLUT5) for gastrointestinal absorption of glucose and fructose may be present in crustaceans, but the invertebrate SGLT-like process functionally appears to resemble a Na^+^/K^+^-dependent SGLT4 more than a Na^+^-dependent SGLT1. However, these functional studies do not presently provide conclusive identity of the transporters described here. Future molecular characterization of sugar transporters in both tissues are needed for a positive identification as well as a description of any apparent differences in cation binding properties that they may display.

## MATERIALS AND METHODS

### Magnesium precipitation technique to purify hepatopancreatic brush border membrane vesicles

Live male lobsters (*Homarus americanus*), weighing approximately 500 g, were purchased from a commercial dealer (Fisherman's Dock, Jacksonville, Florida) and were maintained unfed at 15°C for up to 1 week in an aquarium containing filtered seawater. Hepatopancreatic brush border membrane vesicles (BBMV) of fresh tissue removed from individual lobsters were produced using the MgCl_2_ method of Kessler et al. (Kessler et al., 1978), as modified by Biber at al. (Biber at al., 1981) for mammalian epithelia, and applied to crustacean hepatopancreas (Ahearn et al., 1985).

In this study, an entire hepatopancreas was homogenized for 3 min at high speed in a Waring blender in 60 mL ice-cold Buffer 1 (300 mM mannitol, 5 mM EGTA, 1 mM PMSF, and 12 mM Tris-HCl, pH 7.0) made hypotonic by adding 240 mL ultra-pure water. The homogenate was placed in 8 tubes and centrifuged at 27,000 *g* for 30 min at 4°C. The resulting pellets were combined (pellet 1) and re-suspended in 60 mL ice-cold Buffer 2 (300 mM mannitol, 12 mM Tris/HCl, pH 7.0) and homogenized using 10 strokes of a Potter–Elvehjem tissue grinder at high speed. The homogenate was centrifuged at 27,000 *g* for 30 min at 4°C. The resulting pellet (pellet 2) was re-suspended in 60 mL ice-cold Buffer 2 and homogenized. The homogenate to which 10 mM MgCl_2_ was added was allowed to incubate on ice for 15 min followed by two centrifugations, the first at 3000 *g* for 15 min at 4°C (discard pellet 3) and the second at 27,000 *g* for 30 min at 4°C (save pellet 4). The resulting pellet (pellet 4) was resuspended in 35 mL of buffer 3 (60 mM mannitol, 5 mM EGTA, 12 mM Tris/HCl, pH 7.0) and the MgCl_2_ was repeated on this mixture. Two centrifugations followed, the first at 3000 *g* (discard pellet 5) and the second at 27,000 *g*, retrieving pellet 6 from the second centrifugation. Pellet 6 was resuspended in 35 mL transport buffer (usually 200–300 mM mannitol, 12 mM Hepes, at pH 7.0) and homogenized. This was followed by centrifugation for 30 min at 27,000 *g*. The resulting Pellet 7 was resuspended with a 20 gauge needle in enough transport buffer for experimentation, usually 500–1000 µL.

A small aliquot of this vesicle suspension was used to determine the amount of protein present by the BioRad protein assay (BioRad, Hercules, CA) at 595 nm using a DU 640 spectrophotometer (Beckman Coulter, CO). A standard curve was prepared using various concentrations of bovine serum albumin (BSA). BioRad dye (5 mL) was added to each sample and mixed with a vortexer. The standards were tested using the Bradford–Stahl test, in a spectrophotometer, which yielded absorbances at 595 nm. Three 10 µL samples of the vesicles were tested against the standards. The absorbance values of the vesicles fell on the standard curve and were used to estimate protein concentrations of the vesicles.

Transport experiments were conducted at 23°C using BBMV produced by the method described above and the Millipore filtration technique developed by Hopfer et al. (Hopfer et al., 1973). In these experiments 20 µL of BBMV were added to 180 µL of radiolabeled external medium containing [^3^H]-glucose, [^3^H]-fructose (American Radiochemical Corp., USA) and other chemical constituents specific to each experiment. Incubation of vesicles with the radiolabeled nutrient was continued for time periods from 1 to 60 min after which a known volume of this incubation mixture (20 µL) was withdrawn and plunged into 2 mL ice-cold stop solution (generally an isotonic choline chloride medium) to stop the uptake process. The resulting suspensions were rapidly filtered through Millipore filter paper (0.65 µm) to retain the vesicles and washed with another 3 mL of stop solution. Filters were then added to a Beckman scintillation cocktail and counted for radioactivity in a Beckman LS-6500 scintillation counter. Uptake values were expressed as ρmoles/mg of membrane protein per filter. Each experiment was conducted three times with 3 replicate samples per treatment using membranes prepared from different animals. The data were pooled for subsequent analysis. Values are means ± SEM. Sigma Plot 10.0 curve fitting software (Systat Software, Inc. Point Richmond, CA, USA) was used to present data in figures and to obtain carrier-mediated influx constants.

### Preservation and storage of lobster hepatopancreatic border membrane vesicles (BBMV)

The combined time for hepatopancreatic BBMV isolation and assay is often quite long and the ability to preserve a large quantity of vesicles permits many experiments on the same vesicle population and/or on the same day, thereby facilitating comparison of results. In a previous study, experiments were reported on the preservation of glucose transport and enzyme activities during freezing of BBMV from the intestinal epithelium of the warm-water euryhaline fish, *Oreochromis mossambicus* (Reshkin et al., 1988). Their study showed that the freezing of BBMV in liquid nitrogen preserved the properties of glucose transport found in fresh vesicles, including sodium-dependency, and overshoot characteristics. To assess the effect of freezing on the transport activity of lobster hepatopancreatic BBMV, vesicles prepared from an individual lobster hepatopancreas were loaded with 300 mM mannitol, 12 mM Hepes/Tris at pH 7.0 and the resulting suspension was split into two. One half was placed into liquid nitrogen for 30 min and then stored in −80°C for at least 48 hours. The other half was used immediately to measure 0.1 mM D-glucose uptake over time periods of 15 s, 1, 2, 5, 10, and 60 min of incubation in media consisting of either 150 mM NaCl, KCl or mannitol each with 12 mM Hepes/Tris at pH 7.0. The same experiment was repeated with the frozen half of the BBMV sample.

### *In vitro l*obster intestinal perfusion technique

The part of the intestine used for each experiment was cut from 1 cm posterior to the stomach to about two-thirds of the length of the tail. This fraction of intestine was composed of midgut tissue only. *In vitro* transmural mucosal to serosal (MS) transport of [^3^H]-glucose and [^3^H]-fructose was studied using a perfusion apparatus previously described (Ahearn and Maginniss, 1977). Isolated whole intestine was flushed with physiological saline (410 mM NaCl, 15 mM KCl, 5 mM CaSO_4_, 10 mM MgSO_4_, 5 mM Hepes/KOH at pH 7.1) and mounted on an 18 gauge needle at both ends of the perfusion apparatus using surgical thread. The length and diameter of the experimental intestine were measured and the intestinal surface area was calculated using the equation *A* = π*ld*, where “*l*” and “*d*” represent the length and diameter of the intestine, respectively. The perfusion bath (serosal medium) was filled with 35 mL of physiological saline. The experimental perfusate (the experimental saline plus appropriate experimental treatments) was pumped through the intestine using a peristaltic pump (Instech Laboratories Inc., Plymouth Meeting, PA, USA) at a rate of 0.38 mL min^−1^ [a rate previously shown to provide constant transmural transport in lobster intestine for over 3 h of incubation without added oxygen at 23°C (Conrad and Ahearn, 2005)].

Transport time course experiments were conducted by adding concentrations of D-glucose or D-fructose, and NaCl or KCl to different 50 mL tubes (Falcon, Newark, N.J) containing physiological saline and [^3^H]-glucose or [^3^H]-fructose. Prior to the start of experimentation, triplicate aliquots of each experimental perfusate (200 µL) were collected from each Falcon tube to determine the total counts of radioactively labeled sugar in each tube, and from the bath to determine the amount of background radioactivity at the beginning of an experiment. Experimental solutions were then perfused through the intestine for a total of 30 min. All experimental procedures were carried out at 23°C. Triplicate radioactive samples (200 µL) were collected from the serosal medium after passage across the intestine every 5 min for the duration of each experimental treatment. An equal amount of physiological saline was added to the serosal medium in order to maintain a constant volume in the bath.

The radioactive experimental samples collected were placed in 7 mL tubes containing 3 mL scintillation cocktail and counted for radioactivity. The mean background count was subtracted from each triplicate sample at each time point. Transmural MS rates were expressed as ρmol/cm^2^× min after correction of periodic bath samples for total bath volume.

The two different experimental techniques used in this paper to define the nature of sugar transport in hepatopancreas and intestine are the result of very different anatomy of the two digestive system organs. Therefore, firm quantitative conclusions cannot be drawn from these different methods, but qualitative comparisons between the tissues are appropriate.
